# Contraceptive implant failures among women using antiretroviral therapy in western Kenya: a retrospective cohort study

**DOI:** 10.12688/gatesopenres.12975.2

**Published:** 2020-01-14

**Authors:** Anne Pfitzer, Jacqueline Wille, Jonesmus Wambua, Stacie C Stender, Molly Strachan, Christine Maricha Ayuyo, Timothy F. Kibidi Muhavi, Valentino Wabwile, Supriya D. Mehta, Elizabeth Sasser

**Affiliations:** 1 Maternal and Child Survival Program/Jhpiego, 1776 Massachusetts Ave, NW Suite 300, Washington, DC, 20036, USA; 2 Jhpiego, Arlington Block, 14 Riverside, Nairobi, Kenya; 3 Jhpiego, Cape Town, South Africa; 4 Independent researcher, Nairobi, Kenya; 5 Center for Health Solutions, Kasuku Road, Nairobi, Kenya; 6 School of Public Health, University of Illinois at Chicago, 1603 W Taylor Street, Chicago, IL, 60612, USA; 7 Jhpiego, Seattle, WA, USA

**Keywords:** Drug interactions, contraceptive implants, contraception, ART, HIV

## Abstract

**Background**: Women living with HIV have the right to choose whether, when and how many children to have. Access to antiretroviral therapy (ART) and contraceptives, including implants, continues to increase in Kenya. Studies have reported drug-drug interactions leading to contraceptive failures among implant users on ART. This retrospective record review aimed to determine unintentional pregnancy rates among women 15-49 years of age, living with HIV and concurrently using implants and ART in western Kenya between 2011 and 2015.

**Methods**: We reviewed charts of women with more than three months of concurrent implant and ART use. Implant failure was defined as implant removal due to pregnancy or birth after implant placement, but prior to scheduled removal date. The incidence of contraceptive failure was calculated by woman-years at risk, assuming a constant rate.

**Results**: Data from 1,152 charts were abstracted, resulting in 1,190 implant and ART combinations. We identified 115 pregnancies, yielding a pregnancy incidence rate of 6.32 (5.27–7.59), with 9.26 among ETG and 4.74 among LNG implant users, respectively. Pregnancy incidence rates did not differ between EFV- and NVP-based regimens (IRR=1.00, CI: 0.71-1.43). No pregnancies were recorded among women on PI-based regimens, whereas pregnancy rates for efavirenz and nevirapine-containing regimens were similar, at 6.41 (4.70–8.73) and 6.44 (5.13–8.07), respectively. Pregnancy rates also differed significantly by implant type, with LNG implant users half as likely to experience pregnancy as ETG implant users (0.51, CI: 0.33-0.79, p>0.01).

**Conclusions**: Our findings highlight the implications of drug-drug interaction on women’s choices for contraception.

## Introduction

All women, regardless of HIV status, have the right to choose whether, when and how many children to have. For women living with HIV (WLHIV), the right to decide not only impacts maternal and infant morbidity and mortality but is a pillar of prevention of mother-to-child transmission (PMTCT) by decreasing unintended pregnancies.

Long-acting reversible contraceptives (LARCs), including progestin-only implants, are efficacious, cost-effective, and have high continuation rates. Implants are 99% effective in preventing pregnancy and are increasing in popularity worldwide ^1– 3^. Coupled with UNAIDS’s ambitious goal of reaching 90% of people living with HIV with antiretroviral therapy (ART) by 2020 ^4^, a growing number of women are accessing contraceptives and ART concurrently. The WHO provides medical eligibility guidelines for women using ART ^5^.

Contraceptive prevalence, including implant use, has rapidly increased in Kenya ^6^. Among all women of reproductive age, 15% use implants ^7^. A study found that Kenyan WLHIV were more likely than their non-HIV affected peers to desire no more children and slightly more WLHIV used contraception ^8^. Another study found high overall contraceptive use (91%) among women attending HIV care clinics, but low concurrent use of condoms ^9^.

In Kenya, 5.9% of adults 15–49 years of age were living with HIV in 2015. Western counties had the highest HIV prevalence rates, ranging from 6.7% in Busia to 26.0% in Homa Bay ^10^. HIV treatment became widely available in the public sector in 2005. Until July 2018, WHO-recommended first-line drug regimens consisted of a combination of two nucleoside reverse transcriptase inhibitors (NRTIs) plus a non-nucleoside reverse transcriptase inhibitor (NNRTI). Kenyan guidelines changed over time, in line with global evidence and recommendations ^11, 12^. In 2014, country guidelines recommended the NRTIs tenofovir (TDF) and lamivudine (3TC) plus the NNRTI efavirenz (EFV) as first-line ^13^. In 2017, approximately 54% of all people living with HIV (PLHIV) on ART in Kenya were taking an EFV-based regimen ^14^.

NVP and EFV are metabolized in the liver via cytochrome P450, as is hormonal contraception. Levonorgestrel (LNG, brand names: Jadelle® and Sino-implant/Levoplant®) and Etonogestrel (ETG, brand names: Implanon®, Nexplanon®) are metabolized by the CYP3A4 enzyme along this pathway ^15^. Pharmacokinetic and retrospective clinical studies have described concomitant use of EFV with implants, although several include small samples of women ^16– 21^. Additional prospective studies have examined the pharmacokinetic effects of ART on hormone levels among implant users and found various degrees of impact on ETG and LNG bioavailability or adverse events, including pregnancy ^22– 25^. A large retrospective clinical record review by Patel *et al.* in Kenya revealed unadjusted pregnancy rates of 5.5 (ETG) and 7.1 (LNG) among 24,560 women using EFV ^26^.

Clinicians serving WLHIV need guidance to appropriately counsel their clients, as misinformation is creating uncertainty about how to describe contraceptive choices to these women ^16, 27^. This study aimed to contribute data to the body of evidence related to contraceptive failures among women who are on ART and use implants that is largely informed by smaller-scale pharmacological studies, with the ultimate goal to improve counseling for WLHIV. The primary aim of this retrospective record review is to determine unintentional pregnancy rates among WLHIV (15–49 years old) concurrently using contraceptive implants and ART in nine facilities in Western Kenya between January 2011 and December 2015. The secondary aim is to describe the characteristics of concurrent implant and ART users with and without implant failures and to explore alternative correlates of method failure.

## Methods

We reviewed charts of all women of reproductive age (15–49 years) who had at least three months of concurrent use of any ART and a contraceptive implant, and who accessed services at a high-volume health facility ^1^ offering comprehensive care for PLHIV. To be included in the analysis, the use of an implant had to occur during any period within the dates of January 2011 to December 2015, and taking place at a time that a woman was also receiving ART. Prior to developing a protocol for the chart review, a feasibility assessment was conducted to: pretest data abstraction tools and processes; determine the degree of integration between HIV and FP services in high-volume facilities; establish whether linking HIV and FP client data was possible; and verify that there were cases of implant failure among ART clients. The investigators then prioritized nine health facilities ^1^ in Western Kenya based on completeness of medical records, data management processes, local HIV prevalence, results from past programs and the lack of fees for family planning services. The investigators also excluded facilities in which a similar study was being conducted by Family AIDS Care and Education Services (FACES). To mitigate potential bias, we trained research assistants prior to the initiation of the study using standard operating procedures including a data abstraction form and we conducted a pilot test during this training.

### Data collection

Between January 2016 and March 2017, eleven research assistants (RAs) reviewed medical charts of all women receiving care at Comprehensive Care Clinics (CCC) for PLHIV in nine public-sector facilities in five counties. RAs retrieved medical records for all female clients who were seen at the CCCs between January 2011 and December 2015 and verified whether they were of reproductive age, on ART, and using a contraceptive implant. Women who did not meet these criteria were excluded from further data abstraction. Data abstracted from women’s medical records included: date of birth, implant type (Implanon/Nexplanon® or ETG, Levoplant® and Jadelle® or LNG), date inserted, date removed, reason for removal, date of enrollment into HIV care, age at enrollment into care, date of ART start, date of stopping ART, ART regimen (multiple entries possible for start, end dates and type of ART regimen), date of transfer or death, notations of pregnancy in records, dates of CD4 count(s), viral load results and date taken, number and date of liver function tests done and results, WHO stage (with date), height and weight (to calculate BMI), tuberculosis (TB) status. Treatment start and end dates, and other concurrent chronic conditions (hepatitis, diabetes, hypertension) with dates of diagnosis and other medications used. Records rarely indicated estimated delivery dates (EDD). Instead, our data simply includes the date of a visit that included any notation of a pregnancy. Frequently, the date also coincides with the date of an implant removal. Two study investigators made regular supervisory visits to each RA and reviewed logs and data entry, comparing them against case files. The study was reviewed and approved by the Johns Hopkins School of Public Health review board (#6073) and the Kenya Medical Research Institute scientific and ethical review committee (#NON-SSC 510).

In cases where RAs were unable to complete full data abstraction from the client’s CCC medical record and regardless of the client or regimen used, they searched other facility records, including case notes from the consulting health care provider, Family Planning Register, PMTCT Register, Antenatal Care (ANC) Register, Logistics Management Information System, and laboratory records. For several clients in four Kisumu County facilities, the study team queried Marie Stopes Kenya’s database of family planning outreach events for subsets of implant clients and shared client FP record numbers with RAs to facilitate searching for missing information in facility files or registers.

The investigators sought to identify possible pregnancies due to contraceptive implant failures from medical records: specifically, records of the client’s reason for implant removal as pregnancy or that the client gave birth after receiving an implant and before its removal date.

Each CCC client receives a unique identifier (eleven-digit, alpha-numeric code) at enrollment in HIV care and treatment services. The investigators only used this number to locate client records for data verification purposes. Client names were tallied but not included in data abstraction. De-identified data were entered into REDCap v6.14.0 (Research Electronic Data Capture), a web-based data management application, with limited, password-protected access. RAs were strictly instructed to properly store all paper and electronic copies of records with client-identifying information. Portable electronic devices did not contain identifiable information.

### Data analysis

We estimated contraception failure rates for LNG and ETG implants with concurrent ART regimens containing EFV, NVP, or a protease inhibitor (PI), assuming a constant rate of implant failure. We estimated the incidence of pregnancy per 100-woman years at risk. We defined person years at risk as the start of concurrent implant and ART use (beginning from the start date of whichever was introduced second) to either pregnancy, date of implant removal, end of approved implant effectiveness, end of ART use, or end of the study period, whichever came first.

We used Poisson regression to calculate the incidence rate ratios (IRRs) of pregnancy by age, CD4 count, BMI, ART regimen, and implant type. We repeated this for all six possible combinations of concurrent ART and implant use. Data on TB treatment and viral load were missing for over 96% of women, precluding analysis of these variables; however, we conducted a separate review of those records indicating TB treatment to verify timelines of TB medication in relation to ART regimen, implant use and any pregnancies. Differences were deemed statistically significant at the p<0.05 level (two-sided test of significance). IRRs were calculated after adjusting for potential clustering within HIV clinics, using cluster-adjusted standard errors ^28, 29^. To test for robustness of results, we reran analyses excluding women aged 35–49. Two separate investigators conducted analyses and cross-checked results; both used STATA with slightly different versions (14 and 15).

Kaplan-Meier failure curves were created to visualize time-to-implant failure for the different ART and implant combinations by using the date of unintended pregnancy as the failure date. We present duration of concurrent ART-implant use for interpretation of Kaplan-Meier results.

## Results

### Data cleaning

RAs abstracted 1,612 records from women concurrently using ART and an implant, under two investigators’ close supervision. Investigators excluded all 208 records from Busia County Referral Hospital due to inconsistencies in RAs’ adherence to standard operating procedures. Subsequently, investigators removed records which were missing critical data indicators, including: 36 suspected duplicate records, 50 records with invalid ART or missing implant data, 81 records which indicated that there was fewer than three consecutive months of concurrent implant and ART use, and 85 records where the woman did not meet other inclusion criteria.

All observations without concurrence end dates were censored at December 31, 2015 or earlier if the end of approved implant effectiveness (3 years for Implanon/Nexplanon® and Levoplant® and 5 years for Jadelle®, per WHO prequalifications ^30^) preceded the end of the observation period. Records which listed emtricitabine (FTC) within the ART regimen were regrouped with 3TC due to pharmacokinetic similarity ^31^.

With exclusions, the dataset included 1,152 individual women ( Table 1). The dataset was then expanded so that women who switched to a new ART and/or implant over the course of the study period were recoded as separate observations for each unique ART/implant co-administration, giving a final dataset of 1,190 observations.

**Table 1.** Concurrent use of implants and antiretrovirals by women.

Variable	Women N=1,152	%
1 ^st^ ART regimen during concurrent use		
NVP regimen	365	31.7
EFV regimen	769	66.8
PI regimen	18	1.6
2 ^nd^ ART regimen during concurrent use		
NVP regimen	7	0.6
EFV regimen	16	1.4
PI regimen	9	0.8
No 2nd ART regimen	1,120	97.2
3 ^nd^ ART regimen during concurrent use		
NVP regimen	1	0.1
EFV regimen	0	0.0
PI regimen	1	0.1
No 3rd ART regimen	1,150	99.8
1 ^st^ Implant during concurrence		
ETG	491	42.6
LNG	661	57.4
2 ^nd^ Implant during concurrence		
ETG	2	0.2
LNG	2	0.2
No 2nd implant	1,148	99.7
Facility		
Awendo Sub-County Hospital	229	19.9
Kendu Seventh Day Adventist Hospital,	96	8.3
Kendu Sub-County Hospital	185	16.1
Kisumu Sub-County Hospital	160	13.9
Migosi Health Centre	116	10.1
Nyalunya Health Centre	80	6.9
Railways Dispensary	133	11.6
Siaya County Referral Hospital	153	13.3

ART, antiretroviral therapy; NVP, Nevirapine; EFV, efavirenz; PI, protease inhibitor; ETG, Etonogestrel; LNG, Levonorgestrel.

### Demographic and ART information

Table 2 provides age and clinical status of women during each instance of concurrent use of ART and an implant, which allows individuals who changed their regimen and/or implant during the study period to be counted as more than one observation. The most common combination of implant and ART use was LNG-EFV (39.4%), followed by ETG-EFV (26.7%), LNG-NVP (16.7%), ETG-NVP (14.8%), LNG-PI (1.3%), and ETG-PI (1.0%).

**Table 2.** Age and clinical status of women at the start of each observation of each co-administration.

Variable	Co-administration N=1,190	%
Age at time of combination start		
15 – 24	250	21.0
25 – 34	760	63.9
35 – 49	180	15.1
CD4 count within +/- 1 yr of combination start		
0 – 349	326	27.4
>=350	410	34.5
Missing	454	38.2
BMI within +/- 1 year of combination start		
<18.5	98	8.2
18.5 – 25	770	64.7
25 – 30	178	15.0
>=30	46	3.9
Missing	98	8.2
ART Regimen		
NVP regimen	375	31.5
EFV regimen	787	66.1
PI regimen	28	2.4
Implant		
ETG	506	42.5
LNG	684	57.5
Implant & ART combination		
ETG-NVP	176	14.8
ETG-EFV	318	26.7
ETG-PI	12	1.0
LNG-NVP	199	16.7
LNG-EFV	469	39.4
LNG-PI	16	1.3

BMI: body mass index, ART: antiretroviral therapy, NVP: Nevirapine, EFV: efavirenz, NNRTI: non-nucleoside reverse transcriptase inhibitor, ETG: Etonogestrel, LNG: Levonorgestrel

In total, 32 of the 1,152 women included in the dataset switched ART regimens while using an implant during the study period; 30 women were exposed to two regimens and two women were exposed to three regimens. Four women used implants twice. Three women were exposed to three different combinations of implant and ART. Our sample skews towards shorter periods of co-administration, associated with a recent rise in implant use in Kenya (not shown). The median duration of implant and ART co-administration in our dataset is 1.33 years (not shown). In total, 38 women had multiple observations included in the analysis. The mean time included in first observations was 560 days (median: 488 days); 501 days for second observations (median: 481) and 431 days for third observations (median: 403).

### Tuberculosis and treatment

Table 1 and Table 2 do not show the number of women whose records indicated TB co-infection and treatment during periods of co-administration of implants and ART. We excluded this data point in the regression analyses because of the high proportion of records with no TB data. However, we reviewed the subset of women with records of TB treatment and describe the findings in Box 1.

**Box 1. Exploration of records of women who received treatment for tuberculosis**

As noted, very few of the records had any notations regarding TB status. Clinical records of 45 women (52 co-administrations) indicated TB treatment. Among these, five had pregnancies. However, for two women, documentation indicated pregnancy occurred over 2 years after the completion of TB treatment.

For three women, the possibility of additional drug interactions with rifampicin cannot be ruled out. The documented pregnancies occurred within 3 to 9 months after end of treatment for TB. However, all women were using an EFV-based regimen, so this exploration does not provide any further explanation of why so many women on NVP-based regimen experienced contraceptive failures.

### Pregnancy incidence

Table 3 presents pregnancy IRRs and incidence in person-years of observations broken down by clinical status of women at each observation of co-administration. There were 115 pregnancies in the 1,190 instances of co-administration, yielding a pregnancy incidence rate of 6.32 (95% CI: 5.27–7.59), with 9.26 (95% CI: 7.18–11.96) among ETG and 4.74 (95% CI: 3.65–6.16) among LNG implant users, respectively.

**Table 3.** Pregnancy incidence rates and incidence rate ratios by clinical characteristics.

Variable	Number of pregnancies	Total person-years of concurrence	Pregnancy Incidence Rate ^‡^ (95% CI)	Adj Pregnancy Incidence Rate Ratio (95% CI) ^†^	p-value
Overall (N=1,190)	115	1,818.7	6.32 (5.27 – 7.59)		
Age at time of co-administration start					
15 – 24	30	299.9	10.0 (6.99 – 14.30)	1	
25 – 34	75	1,223.4	6.13 (4.89 – 7.69)	0.61 (0.35 – 1.06)	0.08
35 – 49	10	295.4	3.39 (1.82 – 6.29)	0.34 (0.18 – 0.62)	<0.01 *
CD4 count within +/- 1 year of combination start					
0 -349	42	604.4	6.95 (5.14 – 9.40)	1	
>=350	44	651.9	6.75 (5.02 – 9.07)	0.97 (0.61 – 1.54)	0.90
Missing	29	562.4	5.16 (3.58 – 7.42)	0.74 (0.47 – 1.17)	0.20
BMI within +/- 1 year of combination start					
<18.5	13	149.3	8.71 (5.06 – 15.00)	1.33 (0.67 – 2.65)	0.42
18.5 – 25	80	1,220.0	6.56 (5.27 – 8.16)	1	
25 – 30	18	269.8	6.67 (4.20 – 10.59)	1.02 (0.71 – 1.46)	0.93
>=30	1	65.0	1.54 (0.22 – 10.92)	0.23 (0.02 – 2.24)	0.21
Missing	3	114.6	2.62 (0.84 – 8.12)	0.40 (0.10 – 1.57)	0.19
ART Regimen					
NVP	40	624.4	6.41 (4.70 – 8.73)	1	
EFV	75	1,164.9	6.44 (5.13 – 8.07)	1.00 (0.71 - 1.43)	0.98
PI	0	29.3	0	--	<0.01 *
Implant type					
ETG	59	636.9	9.26 (7.18 – 11.96)	1	
LNG	56	1,181.8	4.74 (3.65 – 6.16)	0.51 (0.33 – 0.79)	<0.01 *
Implant & ART combination					
ETG-NVP	22	253.5	8.68 (5.71 – 13.18)	1	
ETG-EFV	37	369.8	10.00 (7.25 – 13.81)	1.15 (0.72 - 1.84)	0.55
ETG-PI	0	13.5	0	--	<0.01 *
LNG-NVP	18	370.9	4.85 (3.06 – 7.70)	0.56 (0.32 – 0.98)	0.04 *
LNG-EFV	38	795.1	4.78 (3.48 – 6.57)	0.55 (0.34 - 0.89)	0.02 *
LNG-PI	0	15.8	0	--	<0.01 *

*Significant at the p<0.05 level. ^**†**^Adjusted for clustering within HIV clinics using cluster-adjusted standard errors. ‡A sensitivity analysis, among women aged 15–34 that censored women 35 years and older instead of 50 years and older, resulted in slightly higher overall pregnancy incidence (6.89 per 100 person-years [CI: 5.69–8.35]). Other results remained similar, except IRRs for LNG-NVP and LNG-EFV combinations compared to ETG-NVP were no longer statistically significant. BMI, body mass index; ART, antiretroviral therapy; NVP, nevirapine; EFV, efavirenz; PI, protease inhibitor; ETG, etonogestrel; LNG, levonorgestrel.

The likelihood of pregnancy decreased with increasing maternal age; the pregnancy IRRs were 0.61 (95% CI: 0.35–1.06) among 25–34 year-olds and 0.34 (95% CI: 0.18–0.62, p<0.01) among women >35 years of age, using 15–24 year-olds as the reference group. A review of the dates of all pregnancies alongside corresponding dates for switching regimen identified two pregnancies that occurred within 136 and 139 days of switching from a NVP-based regimen to an EFV one. Thus, they are categorized as EFV users, but conception might have occurred when using NVP.

### Analysis of incidence by ART regimen and implant type

The pregnancy incidence rates did not differ between EFV- and NVP-based regimens (IRR = 1.00; 95% CI: 0.71–1.43). No pregnancies were recorded among women on PI-based regimens among both ETG and LNG implant users, which was statistically significant. There was a statistically significant difference in pregnancy rates based on implant type, with LNG implant users half as likely to become pregnant than ETG implant users (IRR: 0.51, 95% CI: 0.33–0.79, p<0.01).

Table 3 shows pregnancy incidence rates and pregnancy IRRs for all six implant-ART combinations against the reference of the ETG-NVP combination, which was chosen as reference group based upon previously cited evidence of minimal drug-drug interactions. All combinations except ETG-EFV were significantly different from the reference. Similarly, a separate analysis of LNG-ART combinations revealed no statistical difference between the adjusted pregnancy IRR for LNG-NVP compared to a reference of LNG-EFV (1.015). The IRR for LNG-PI remained significant compared to LNG-EFV.

**Figure 1.**
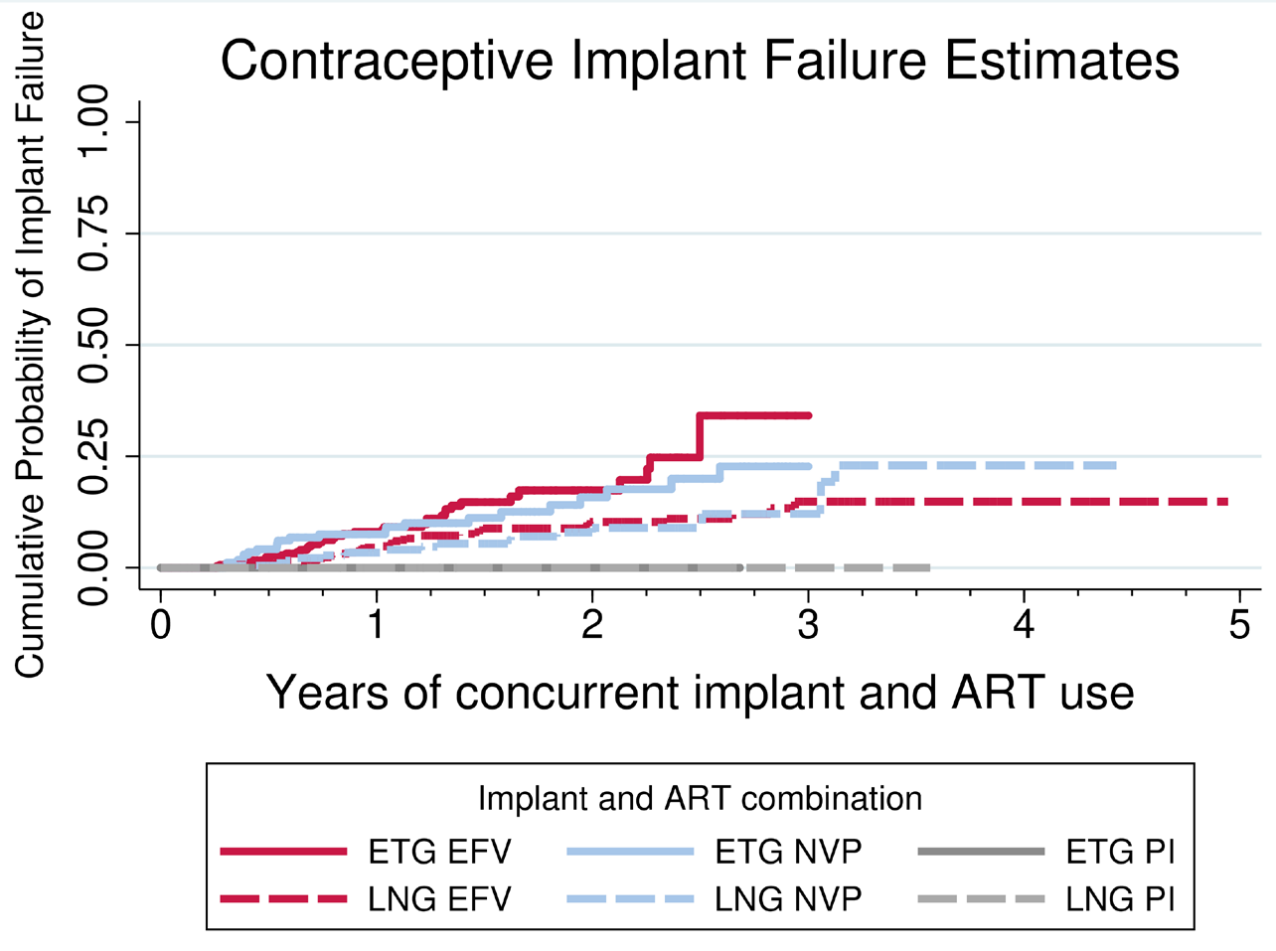
Contraceptive implant failure estimates.

ART, antiretroviral therapy; NVP, nevirapine; EFV, efavirenz; ETG, etonogestrel; LNG, levonorgestrel; PI, protease inhibitor.

Duration to failure curves ( Figure 1) show that pregnancies began occurring within months of concurrent use, steadily accumulating thereafter. The shortest time to pregnancy was 94 days (16.5% and 48.7% within 6 and 12 months, respectively). ETG implant users had more early pregnancies than LNG implant users, but this is likely correlated with higher overall pregnancy incidence and shorter approved duration of use.

The sensitivity analysis (see footnote in Table 3) suggests a lack of robustness in contraceptive failure rate differences across drug-drug combinations by type of implant, a likely artifact of higher use of ETG implants by younger women.

## Discussion

An increase in the incidence of pregnancy among implant users on ART may negatively impact acceptability and trust in implants as a contraceptive choice for all women and their partners, regardless of HIV status. Our findings confirm earlier reports of implant failures among women taking EFV-based ART, with an incidence rate of 4.78 per 100-person years for LNG and 10.00 per 100-person years for ETG implants. However, pregnancy incidence rates were considerably higher than found in the general population or that we had hypothesized at the start of the study ^32, 33^. Our results differ from previous studies in that NVP use was also associated with a high incidence of pregnancies ^24, 34, 35^. Among NVP users, pregnancy incidence rates were similar to EFV users, 4.85 per 100-person years and 8.68 per 100-person years with concurrent LNG and ETG users, respectively. This is the first report, to our knowledge, to present evidence that NVP-based ART regimens may also influence effectiveness of both ETG and LNG. We explored the data in multiple ways, including visually reviewing the records of women who experienced a pregnancy concurrent with NVP use and an implant, without finding any patterns that would explain this finding other than potential pharmacokinetic interactions between NVP and other drug classes metabolized by the cytochrome P450 system – LNG and ENG are both metabolized by CYP3A4 ( https://aidsinfo.nih.gov/drugs/116/nevirapine/17/professional#Section_7) ^34^. However, neither Scarsi *et al*. ^24^ nor Chappell *et al*. ^35^ demonstrated that co-administration of LNG and NVP impacted phamacokinetic efficacy outcomes. Future advances in pharmacogenomics could potential uncover reasons why subsets of women experience unexpected drug-drug interactions ( https://www.statnews.com/2018/10/24/precision-medicine-contraceptive-choice/). Co-administration with other medications that induce the P450 enzymes may contribute to the finding; however, we could not assess this in detail, other than to review of documentation of TB treatment in clinical records. The nearly equivalent rate of implant failures among NVP and EFV users may give indication of a higher ‘baseline’ rate of failures, however we are unable to determine this, as we only reviewed efficacy of implants among WLHIV on ART.

Our exclusion of any pregnancy detected within three months of initiating co-administration minimizes accidental insertions of implants when conception has already occurred, which was a common problem in the Australia post-marketing study and could explain pregnancies in other studies ^36^. Inclusion of all observations linked with any co-administration of ART and implants might have generated even higher pregnancy incidence rates. In our study, pregnancy incidence rates in women using EFV were higher for women using ETG implants than those using LNG implants, but this finding is not robust among younger women. This pattern was repeated among NVP users.

### Limitations

Retrospective studies can be limited due to issues of reliability and completeness of medical charts, as well as possible bias and confounding in how data are abstracted. Prospective studies with more frequent and standardized timing of follow-up could address some of these limitations. Prospectively capturing data could also allow for further analysis of drug-drug interactions with either ART or implants, such as rifampicin for TB or artemether-lumefantrine for malaria. However, the necessary financial investment and length of time would be substantial.

Our retrospective chart review had intended to measure multiple factors, including stage of HIV disease and other drug-drug interactions; however, incompleteness of medical records did not allow for adequate analysis to answer these questions. The absence of documented TB status in the great majority of records was disappointing given high prevalence of co-morbidity with HIV. Only 45 records documented TB treatment during the study period, some of which documented treatment completion in 2010 prior to the study period or after a pregnancy. However, three pregnancies in our dataset were possibly due to a triple drug interaction, all of which involved an EFV-based regimen, consistent with guidelines. While null TB test findings are potentially omitted from records more frequently than active TB or treatment, we cannot eliminate the possibility of additional drug interactions with rifampicin or other medications. We should also note unclear definition of pregnancies in our data, linked with lack of details in medical records. We had also hoped to draw conclusions about timing of failure over time of implant use, in case an earlier removal and replacement of the implant could address the problem of drug-drug interactions. However, because of recent expansion in use of implants in Western Kenya, most of the implant users in our data set had relatively a short duration of concurrent use with ART.

Biases may have also been introduced to the results because of variations in how RAs assessed abstracted records, due to the differing record management systems in the study facilities. How pregnancy was recorded also was not consistent across all charts or sites, with some uncertainty as to whether recorded and thus abstracted dates of pregnancies referred to the date a pregnancy was confirmed or rather the estimated date of delivery (EDDs). While we sought to abstract EDDs, we did not require RAs to calculate EDDs if the only information was that pregnancy was confirmed at a single point in time. Given the limitations in completeness of charts in the CCCs, the RAs diligently sought evidence of pregnancies in other units of the facilities, but it is difficult to do this systematically across diverse facilities.

### Implications for practice

Women should be afforded choices when selecting FP and ART. In 2016, WHO included dolutegravir (DTG) as an alternative drug for first-line regimens, which became available in Kenya in 2017 ^37^ and has been recommended by WHO as first-line treatment in combination with tenofovir and lamivudine since December 2018. NVP is no longer recommended, and EFV will remain as an alternative to DTG in the first line regimen, consistent with recent WHO guidelines ^38^, particularly for women of reproductive age intending to become pregnant or at risk of pregnancy, given concerns about potential neural tube defects ^12^. Co-administration with implants has not been studied, although an interaction is unlikely, as DTG does not inhibit or induce CYP450 enzymes ^16^. In one small study DTG had no impact on norgestimate and ethinyl estradiol levels ^39^. Results from a study in Botswana by Westhoff to assess change in ETG plasma levels among ETG implant users taking DTG are expected in late 2019 ^40, 41^ and a study in Kenya by Patel will assess the pharmacodynamics of both ETG and LNG implants use concurrent with DTG or a lower dose EFV regimen ^42^.

It remains challenging to incorporate the results of this study into service delivery for WLHIV. It is premature to discourage women from adopting implants. However, women on NNRTIs need information, in simple, yet accurate language, about the drug-drug interaction and possibility of method failure. With scale up of transition to DTG and nearly 4 million already on this drug, this becomes less important. ( https://medicinespatentpool.org/mpp-media-post/five-years-on-3-9-million-people-in-the-developing-world-have-access-to-hiv-treatment-dolutegravir-thanks-to-access-oriented-voluntary-licensing-agreements/). Experiences in South Africa have demonstrated the difficulties in ensuring providers understand these drug-drug interactions and are comfortable sharing information with clients ^27^. One option is to refer to the tier of effectiveness ^43^, but explain that for women on certain ART regimens, the implant falls somewhere in the middle tier of effectiveness: slightly better than pills or injectables, but less effective than IUDs or sterilization. Regardless of regimen, women should retain the right to make fully informed decisions about which contraceptive option works best for them and what level of method failure risk is acceptable, given that they cannot, under current HIV guidelines, switch ART regimen based on their fertility preferences. Ideally, building a client-centered culture would allow WLHIV desiring control over their fertility to be offered DTG concurrently with an implant or other LARC.

At minimum, programs should encourage better documentation of all medications in medical charts. Health systems should establish mechanisms to track adverse events (pregnancies) in WLHIV using ART. Study facilities had ongoing efforts to integrate FP within HIV care and treatments services. Fully integrating FP services by including LARCs within services for WLHIV may improve care. Health systems should also ensure that IUDs are as accessible as implants and remove barriers to sterilization services. Research into the benefits and costs of alternative service delivery models could inform national policies.

In conclusion, implants are highly effective; however, clients using a NNRTI-containing ART regimen need additional information about higher incidence of pregnancies when used in combination with ARVs to allow them to make informed decisions about contraceptive options.

## Data availability

### Underlying data

The dataset analyzed for this study was generated from client medical records under ownership of the Kenyan Ministry of Health. The authors’ permission to study this data does not extend to publically sharing the full dataset without prior permissions from the Kenyan Ministry of Health. Access to the de-identified dataset may be obtained by submitting a request to the Kenyan Medical Research Institute (KEMRI) ( seru@kemri.org) and the Jhpiego Open Data Help team ( OpenDataHelp@jhpiego.org), copied to anne.pfitzer@jhpiego.org, with a detailed description of the intended use and an IRB-approved protocol for secondary data analysis. Data will be provided under the condition that researchers have provided the required permissions from the Kenyan National AIDS Control Program and KEMRI.

## References

[1] Polis CB Bradley SE Bankole A: Typical-use contraceptive failure rates in 43 countries with Demographic and Health Survey data: summary of a detailed report. *Contraception.* 2016;94(1):11–17. [cited 2018 Aug 21]. 10.1016/j.contraception.2016.03.011 27018154 PMC4970461

[2] Christofield M Lacoste M: Accessible Contraceptive Implant Removal Services: An Essential Element of Quality Service Delivery and Scale-Up. *Glob Health Sci Pract.* 2016;4(3):366–372. 10.9745/GHSP-D-16-00096 27577239 PMC5042693

[3] Jacobstein R: Liftoff: The Blossoming of Contraceptive Implant Use in Africa. *Glob Health Sci Pract.* 2018;6(1):17–39. 10.9745/GHSP-D-17-00396 29559495 PMC5878070

[4] UNAIDS: 90-90-90: An ambitious treatment target to help end the AIDS epidemic.Geneva, Switzerland: UNAIDS;2014 Reference Source

[5] World Health Organization: Medical eligibility criteria for contraceptive use: Fifth edition.Geneva, Switzerland: World Health Organization;2015 Reference Source

[6] Kenya Demographic Health Survey.Nairobi, Kenya: Kenya National Bureau of Statistics; Kenya Ministry of Health; Kenya National AIDS Control Council; Kenya Medical Research Institute; National Council for Population and Development; ICF International;2015 Reference Source

[7] PMA-2020 Datalab: Performance Monitoring and Accountability 2020. [cited 2017 Nov 16]. Reference Source

[8] Kimani J Warren C Abuya T: Family planning use and fertility desires among women living with HIV in Kenya. *BMC Public Health.* 2015;15(1):909. [cited 2017 Nov 16]. 10.1186/s12889-015-2218-z 26381120 PMC4574729

[9] Antelman G Medley A Mbatia R: Pregnancy desire and dual method contraceptive use among people living with HIV attending clinical care in Kenya, Namibia and Tanzania. *J Fam Plann Reprod Health Care.* 2015;41(1):e1. [cited 2017 Nov 16]. 10.1136/jfprhc-2013-100784 25512359 PMC4664147

[10] HIV situation in Kenya - Open Data Blog. [cited 2018 Aug 21]. Reference Source

[11] Ministry of Health. Guidelines for antiretroviral therapy in Kenya. Nairobi: Kenya National AIDS and STI Control Programme 2011. Reference Source

[12] World Health Organization. Interim Guidelines: Updated Recommendations on first-line and second-line antiretroviral regimens and post-exposure prophylaxis and recommendations on early infant diagnosis of HIV:interim guidelines. Supplement to the 2016 consolidated guidelines on the use of antiretroviral drugs for treating and preventing HIV infection. Geneva: World Health Organization; 2018 (WHO/CDS/HIV/18.51). Licence: CC BY-NC-SA 3.0 IGO.2018 Reference Source

[13] Ministry of Health; National AIDS and STI Control Program (NASCOP). Guidelines on Use of Antiretroviral Drugs for Treating and Preventing HIV Infection: A rapid advice. 2014 Reference Source

[14] Kenya Anti-Retroviral medicines (ARVs) Stock Situation.Nairobi, Kenya: Kenyan Ministry of Health, NASCOP Unit;2017.

[15] Smith PF DiCenzo R Morse GD: Clinical pharmacokinetics of non-nucleoside reverse transcriptase inhibitors. *Clin Pharmacokinet.* 2001;40(12):893–905. 10.2165/00003088-200140120-00002 11735608

[16] Nanda K Stuart GS Robinson J: Drug interactions between hormonal contraceptives and antiretrovirals. *AIDS.* 2017;31(7):917–952. [cited 2018 May 24]. 10.1097/QAD.0000000000001392 28060009 PMC5378006

[17] Matiluko AA Soundararjan L Hogston P: Early contraceptive failure of Implanon in an HIV-seropositive patient on triple antiretroviral therapy with zidovudine, lamivudine and efavirenz. *J Fam Plann Reprod Health Care.* 2007;33(4):277–278. 10.1783/147118907782101724 17925115

[18] Lakhi N Govind A: Implanon ® failure in patients on antiretroviral medication: the importance of disclosure. *J Fam Plann Reprod Health Care.* 2010;36(3):181–2. 10.1783/147118910791749164 20659385

[19] McCarty EJ Keane H Quinn K: Implanon® failure in an HIV-positive woman on antiretroviral therapy resulting in two ectopic pregnancies. *Int J STD AIDS.* 2011;22(7):413–414. [cited 2017 Nov 16]. 10.1258/ijsa.2009.009469 21729965

[20] Leticee N Viard JP Yamgnane A: Contraceptive failure of etonogestrel implant in patients treated with antiretrovirals including efavirenz. *Contraception.* 2012;85(4):425–427. [cited 2017 Nov 16]. 10.1016/j.contraception.2011.09.005 22036046

[21] Sevinsky H Eley T Persson A: The effect of efavirenz on the pharmacokinetics of an oral contraceptive containing ethinyl estradiol and norgestimate in healthy HIV-negative women. *Antivir Ther.* 2011;16(2):149–156. 10.3851/IMP1725 21447863

[22] Vieira CS Bahamondes MV de Souza RM: Effect of antiretroviral therapy including lopinavir/ritonavir or efavirenz on etonogestrel-releasing implant pharmacokinetics in HIV-positive women. *J Acquir Immune Defic Syndr.* 2014;66(4):378–385. 10.1097/QAI.0000000000000189 24798768

[23] Kreitchmann R Innocente AP Preussler GMI: Safety and efficacy of contraceptive implants for HIV-infected women in Porto Alegre, Brazil. *Int J Gynecol Obstet.* 2012;117(1):81–82. 10.1016/j.ijgo.2011.12.002 22249127

[24] Scarsi KK Darin KM Nakalema S: Unintended Pregnancies Observed With Combined Use of the Levonorgestrel Contraceptive Implant and Efavirenz-based Antiretroviral Therapy: A Three-Arm Pharmacokinetic Evaluation Over 48 Weeks. *Clin Inf Dis.* 2016;62(6):675–682. 10.1093/cid/civ1001 26646680 PMC4772838

[25] Perry SH Swamy P Preidis GA: Implementing the Jadelle implant for women living with HIV in a resource-limited setting: concerns for drug interactions leading to unintended pregnancies. *AIDS.* 2014;28(5):791–793. 10.1097/QAD.0000000000000177 24401645

[26] Patel RC Onono M Gandhi M: Pregnancy rates in HIV-positive women using contraceptives and efavirenz-based or nevirapine-based antiretroviral therapy in Kenya: a retrospective cohort study. *Lancet HIV.* 2015;2(11):e474–e482. [cited 2017 Nov 17]. 10.1016/S2352-3018(15)00184-8 26520927 PMC4632202

[27] Patel RC Morroni C Scarsi KK: Concomitant contraceptive implant and efavirenz use in women living with HIV: perspectives on current evidence and policy implications for family planning and HIV treatment guidelines. *J Int AIDS Soc.* 2017;20(1):21396. [cited 2018 Aug 21]. 10.7448/IAS.20.1.21396 28530033 PMC5515020

[28] Williams RL: A note on robust variance estimation for cluster-correlated data. *Biometrics.* 2000;56(2):645–646. 10.1111/j.0006-341X.2000.00645.x 10877330

[29] Rogers WH: Regression standard errors in clustered samples. *Stata Tech Bull.* 1993; STB-13. Reference Source

[30] Medicines/Finished Pharmaceutical Products | WHO - Prequalification of Medicines Programme. [cited 2018 Aug 21]. Reference Source

[31] Ford N Shubber Z Hill A: Comparative efficacy of Lamivudine and emtricitabine: a systematic review and meta-analysis of randomized trials. Wainberg M, editor. *PLoS One.* 2013;8(11):e79981. [cited 2018 Aug 21]. 10.1371/journal.pone.0079981 24244586 PMC3823593

[32] Implanon NXT Product Information. CCDS-MK8415-IPTx-032014. A14909.2016 Reference Source

[33] Sivin I Nash H Waldman S: Jadelle levonorgestrel rod implants: a summary of scientific data and lessons learned from programmatic experience. New York, NY: Population Council;2002 Reference Source

[34] Russo G Paganotti GM Soeria-Atmadja S: Pharmacogenetics of non-nucleoside reverse transcriptase inhibitors (NNRTIs) in resource-limited settings: Influence on antiretroviral therapy response and concomitant anti-tubercular, antimalarial and contraceptive treatments. *Infect Genet Evol.* 2016;37:192–207. [cited 2018 Aug 21]. 10.1016/j.meegid.2015.11.014 26602158

[35] Chappell CA Lamorde M Nakalema S: Efavirenz decreases etonogestrel exposure: a pharmacokinetic evaluation of implantable contraception with antiretroviral therapy. *AIDS.* 2017;31(14):1965–1972. 10.1097/QAD.0000000000001591 28692531 PMC5578871

[36] Harrison-Woolrych M Hill R: Unintended pregnancies with the etonogestrel implant (Implanon): a case series from postmarketing experience in Australia. *Contraception.* 2005;71(4):306–308. [cited 2018 Aug 21]. 10.1016/j.contraception.2004.10.005 15792651

[37] Kenya to introduce better treatment for people living with HIV. Unitaid. [cited 2018 Aug 21]. Reference Source

[38] WHO. Update of recommendations on first- and second-line antiretroviral regimens. Geneva, Switzerland: World Health Organization; 2019 (WHO/CDS/HIV/19.15). Licence: CC BY-NC-SA 3.0 IGO. Reference Source

[39] Song IH Borland J Chen S: Dolutegravir Has No Effect on the Pharmacokinetics of Oral Contraceptives With Norgestimate and Ethinyl Estradiol. *Ann Pharmacother.* 2015;49(7):784–789. 10.1177/1060028015580637 25862012 PMC4472613

[40] Effect of Dolutegravir on Etonogestrel Levels in HIV-infected Women in Botswana. ClinicalTrials.gov. [cited 2018 Aug 21]. Reference Source

[41] Organization WH: Policy brief: updated recommendations on first-line and second-line antiretroviral regimens and post-exposure prophylaxis and recommendations on early infant diagnosis of HIV: HIV treatment, interim guidance. World Health Organization;2018 Reference Source

[42] A pharmacokinetic evaluation of new first-line antiretroviral therapy regimens and contraceptive implants among HIV-positive women in Kenya. NIH Research Portfolio Online Reporting Tools (RePORT) Project number 5R21AI136645-02 [ projectreporter.nih.gov]. Reference Source

[43] Stanback J Steiner M Dorflinger L: WHO Tiered-Effectiveness Counseling Is Rights-Based Family Planning. *Glob Health Sci Pract.* 2015;3(3):352–357. 10.9745/GHSP-D-15-00096 26374797 PMC4570010

