# Supplementary material for: Contraceptive implant failures among women using antiretroviral therapy in western Kenya: a retrospective cohort study
Source: Gates Open Res. 2020 Jan 14;3:1482. Originally published 2019 Jun 4. [Version 2] doi: 10.12688/gatesopenres.12975.2 (PMC6996231; doi:10.12688/gatesopenres.12975.2)
Supplement: Peer review history

# Peer review: Reviewer response for version 2

**DOI:** 10.21956/gatesopenres.14263.r28448

**Published:** 2020-02-05

**Authors:** Rena Patel

**Affiliations:** 1 Department of Medicine, University of Washington, Seattle, WA, USA

The authors have made marked changes to their manuscript to account for the reviewer comments. The biggest outstanding question still remains: why are your point estimates telling a different story than what has been reported, admittedly in limited studies, to date regarding the implant failure rates with efavirenz-containing vs. other ART?  What this requires is for you to think through what your potential sources of bias are (which you have done) and in which direction those biases would push your point estimates, if any (e.g. differential vs. non-differential misclassification).

I confirm that I have read this submission and believe that I have an appropriate level of expertise to confirm that it is of an acceptable scientific standard.

# Peer review: Reviewer response for version 2

**DOI:** 10.21956/gatesopenres.14263.r28449

**Published:** 2020-01-31

**Authors:** Kavita Nanda

**Affiliations:** 1 Contraceptive Technology Innovation, Global Health, Population and Nutrition, FHI360, Durham, NC, USA

The authors have made marked changes to their manuscript to account for the reviewer comments. The biggest outstanding question still remains: why your point estimates telling a different story than what has been reported, admittedly in limited studies, to date regarding the implant failure rates with efavirenz-containing vs. other ART? What this requires is for you to think through what your potential sources of bias are (which you have done) and in which direction those biases would push your point estimates, if any (e.g. differential vs. non-differential misclassification).

I confirm that I have read this submission and believe that I have an appropriate level of expertise to confirm that it is of an acceptable scientific standard.

# Peer review: Reviewer response for version 1

**DOI:** 10.21956/gatesopenres.14082.r27381

**Published:** 2019-08-14

**Authors:** Rena Patel

**Affiliations:** 1 Department of Medicine, University of Washington, Seattle, WA, USA

**Review**

 The authors present an important analysis related to possible higher contraceptive failures with specific concomitant antiretroviral therapies (ART). They undertook a retrospective chart review in their affiliated health facilities in western Kenya to help determine if contraceptive implants and efavirenz (EFV)-containing ART are implicated in possible higher failures, as has been shown by some prior data. Overall, the work is adequately presented though there are several clarifications, some more major than minor, that they could provide, especially in the methods and results sections (e.g. justification for choice of reference categories), to aid others in better replicating or interpreting their results. Another major concern is that the authors provide a very cursory interpretation of their findings, and given that they differ from the already published data to date, this is one of the key questions—why are your study findings different?—that should have been elucidated in their discussion.  Another concern is that the authors make several statements, in the introduction and discussion sections (also these sections require some better organization and flow, more accurate citations, etc.), that are difficult to justify based on the leading PK data (my sense is that the authors would have benefited from including even a pharmacology expert in their authorship team, which could be considered even now). Detailed feedback is provided below.

**Abstract**

- Background line: technically these are considered drug-drug interactions, as the term “drug interactions” is non-specific

 
**Introduction**

- The last line in the 2 ^nd ^paragraph is extremely vague; I don’t understand what the authors are trying to convey. It would be better to more explicitly elucidate their thinking.
- The first line in the 3 ^rd ^paragraph should have a citation, and if it is the same citation as #6, then it should be cited with the first sentence with the assumption that the 2 ^nd ^sentence is supported by the same citation (unless the citation standards for this journal indicate otherwise).
- Same paragraph: The 3 ^rd ^sentence needs a bit of fine tuning, in that this combination is generally recommended as 1 ^st ^line ART regimen.  Also, the next line appears inaccurate on the year; if I am not mistaken, single pill combination containing EFV, was the recommended 1 ^st ^line ART regimen before 2015 in Kenya (maybe even before 2012?, and please provide your citation for this assertion if you disagree). Also, the next paragraph doesn’t make a lot of logical sense as written, and if the authors want to name the leading 1 ^st ^line ART regimen, they should name all 3 components of it. The “n” in nevirapine should not be capitalized. For the last line, if they can cite specific number on how many women of reproductive potential were on EFV-containing regimen, the sentence would be more to the point.
- It seems like the paragraphs in the introduction could be organized better. For example, the first line in the 2 ^nd ^paragraph discusses LARCs and implants and then discussion switches to ART for the rest of the paragraph. The 3 ^rd ^paragraph then discusses more on ART and the 4 ^th ^comes back to contraceptives. The 5 ^th ^paragraph then goes into describing the potential pathway for any drug-drug interactions at play here, and the data to date on this topic. Strongly reconsider the organization of the intro section for more logical flow.
- 5 ^th ^paragraph: 2 ^nd ^line, technically 4 of the 6 citations are for case reports (which are not the same as retrospective clinical studies). The line discussing the Uganda PK study starts with “Similarly” which does not make any logic sense. It would greatly help readers if the authors only used either ETG/LNG acronyms or the brand names, but not both. Last sentence, technically this was not a record review, as is the current study, but rather a retrospective study of electronic medical record data, it would help to indicate the denominator for the rates i.e. “per 100 women-years of implant use,” and it appears that citation number #19 is mistakenly included here. 
- 6 ^th ^paragraph: it appears the first line mistakenly cites #13 (as that citation is not applicable to their statement). Arguably, the goal of the 2 ^nd ^and 3 ^rd ^lines are redundant, might consider combining by saying something along the lines of: “With the ultimate goal of improving counseling for WLHIV, the primary aim of this…”

 
**Methods**

- Data collection, 1 ^st ^paragraph: how did you know who was seen between Jan 2011 and Dec 2015? When listing all the variables you extracted, it might make sense to name them all and indicate that the applicable dates were also extracted (instead of mentioning it twice, and presumably it is applicable to not only viral load results but also CD4 count results). It may help to discuss how pregnancies are detected generally in these facilities—i.e. was it detected with clinical presentation while being gravid or via routine biochemical testing (e.g. urine pregnancy tests)?
- 2 ^nd ^paragraph: might consider saying “facilities” throughout the manuscript, unless you do literally mean “hospitals” in which case it would help to explain which hospitals you selected and why.
- Data cleaning paragraphs: There is a lot of detail here not usually included in publications, but the details do help. It appears only 38 women had a 2 ^nd ^or 3 ^rd ^observation in the dataset; it might help to state it as something along these lines.  Given the organization of Table 1, you could consider including here or in the text, the median duration of observation time for 1 ^st ^observation, 2 ^nd ^observation, etc.  Could you provide the justifications to censor observation time after the recommended use of implant durations, given that maintaining those times arguably provides even stronger evaluation for real-world effectiveness (if the woman is still using the implant past its recommended duration)?
- Data analysis: only clarification is on last line of first paragraph, as “x100” is not clear if it applies to numerator or denominator.  Perhaps consider saying “per 100 women-years.”
- A couple questions: How did you arrive at your sample size? was there ever a determination of the required sample size to achieve power for detecting a difference between certain combinations? Or did you simply go with a convenience sample for what was feasible for the study duration? Could you also describe how you determined ascertainment of the incident pregnancy to a combination category? i.e. was it the combination category at the time of the pregnancy detection or did you base it on date of likely conception (and if latter, please describe how you calculated the date of likely conception)? Did you consider double entry for cases where a pregnancy was detected (i.e. some cross-check or validation of your initial data entry), as data entry errors by RAs could have also occurred?

 
**Results**

- Overall, the various comparisons are confusing and it is difficult for me to follow which comparison is being made when. It may help to use additional subheadings to clearly separate the findings, and it may also help to prioritize the main findings first
- Small note on Table 3, that the 3 ^rd ^category of ART is “non-NNRTI” which does not necessarily equate to PI-containing ART (which is how the 3 ^rd ^category is defined in the manuscript), so it would be better to clarify if PI-only. Of note, I would not recommend a non-specific non-NNRTI category as the drug-drug interaction implications vary markedly by not only ART family but individual antiretrovirals.
- Paragraph after Table 3 that starts with “Analysis of implant…”: Could you provide justification for why ETG-EFV combination was used as the referent group? It seems like picking a category where you don’t expect a drug-drug interaction, such as with NVP, is a better “control” group or the counterfactual. (Of note, when looking at Table 3, I see that you used ETG-NVP as your reference category—please rectify the difference.) More importantly, please justify why you would use ETG-EFV as the referent group for the LNG combinations? Wouldn’t you want to compare each implant type against itself, given the possible differences in hormone concentrations, affinity to receptors, recommended duration of use, number of rods, etc.?  As a possible secondary analysis, you can compare the ETG to LNG implants.  Also, the conjunctions of “EFV and NVP or using a PI” are confusing, please consider rephrasing for clarity.
- Figure 1: note use of PI here as opposed to NNRTI
- 2 ^nd ^to last paragraph: it’s not clear the marked utility in Figure 2, however if you wish to keep it, please consider dividing or creating 3 separate figures for, for example, the ART type with then the breakdown of implant type within it. Also, when reporting median duration of co-administration time, please also report standard IQR or range, and consider doing this for your 3 or 6 combination categories.
- Several of the results paragraphs end in interpretation of the findings, which would be better discussed in the discussion section.

 
**Discussion**

- The biggest missed opportunity in the discussion section is in better elucidating why no significant differences were found between EFV and NVP and respective implant combination groups. What are the possible reasons you did not detect a difference when several PK studies and other retrospective studies (namely Perry and Patel) have found differences? What are possible biases that arise from your chosen methodological approach? NVP’s DDI profile is markedly different from EFV, so grouping them together and suggesting that all NNRTIs have similar effects is not well supported by existing PK and clinical studies. To this point, etravirine and rilpivirine, other NNRTIs, also have very different DDI and PK profiles than EFV. Given the topic is deeply rooted in the PK world, greater clarity from the PK perspective on the study findings is a must.
- First paragraph: the use of “ranged” is likely not appropriate, as those are specific point estimates for certain combination categories, correct? So you might as well state that they are for x and y combinations, respectively. Also, generalizing to all NNRTIs is not appropriate as each NNRTI has a very different drug-drug interactions profile. Also, the first paragraph could use some rewording. It seems like in the middle of it, you are trying to highlight that, surprisingly, you found a higher rate of failures with NVP than you had hypothesized at the start of your study. Finally, in the latter half of the first paragraph, the interpretation of the authors for this finding comes across as too definitive—isn’t it also possible that the nearly equivalent rate of implant failures among NVP and EFV users you found may signal possibly a “baseline” rate of failures among implant users? Of course, it does not explain the higher than the “baseline” rate that has been described in other settings.
- First half of second paragraph: indeed, it is robust of you to censor pregnancies within the first 3 months of implant placement in cases where the implant may have been placed with a pregnancy already in place. However, shouldn’t that result in a bias towards a lower pregnancy rate compared to the post-marketing surveillance study from Australia that you are comparing to? It would be prudent to spend some space in the discussion section to offer thoughts on why the overall pregnancy rates are higher in your study that other, predominantly non-African, settings.
- The second half of second paragraph: this is a very interesting finding, and deserves discussion in a separate paragraph—why do you think this difference might exist? Why does it appear to diminish among younger women?
- Implications for practice: 1 ^st ^paragraph, cite your basis for not anticipating DDIs with DTG and implants (i.e. the OC PK study by Song *et. al*. ^1^).  2 ^nd ^paragraph, shouldn’t women also be offered the choice to pick an ART regimen that meets their values and preferences (e.g. DTG, knowing that there is currently an unknown and possible risk with adverse birth outcomes)? Given the great women-centered approach the intro and the first half of this paragraph takes, it seems like a missed opportunity to also not advocate for WLHIV to also be informed with best information available at the moment to make their own decisions.

 
**Throughout**

- Considering saying either “failure rates” or “unintended pregnancy” throughout the manuscript, as switching between the two, often in back to back sentences, is confusing for readers who may not be familiar with the “contraception world” where the two equate each other.

I confirm that I have read this submission and believe that I have an appropriate level of expertise to confirm that it is of an acceptable scientific standard, however I have significant reservations, as outlined above.

## References

[1] : Dolutegravir Has No Effect on the Pharmacokinetics of Oral Contraceptives With Norgestimate and Ethinyl Estradiol. *Ann Pharmacother*.2015;49(7) : 10.1177/1060028015580637 784-9 10.1177/1060028015580637 25862012PMC4472613

# Response

**Published:** 2019-12-21

**Authors:** Anne Pfitzer

**Affiliations:** Jhpiego, USA

Thank you for taking the time to summarize and provide an overall assessment of the paper.

 We note your reservations and have attempted to address them in the paper and in the responses below.

 Regarding the choice of reference group, we have kept our presentation as it was, but added results of modeling within LNG reference in the text. The results remain unchanged for comparisons within ETG-ART combinations.

 We agree that this paper is unable to provide a plausible explanation for some of the findings of drug-drug interactions with NVP-based regimen. We argue that we are merely sharing observations from medical chart data and that it is beyond our scope to assess underlying reasons. We hope that by publishing these data, we encourage further investigations. If this study is a complete outlier, then reviewers will come to their own conclusions, including about the difficulties of using retrospective, poorly documented medical charts for investigations of this sort.

 We have taken your feedback about involving a pharmacologist in the review of our paper and attempted to do this, but could not find anyone able or willing to assist us.

 Abstract:  we have corrected the terminology used for drug-drug interactions and throughout the rest of the manuscript.

 Introduction: We accepted your feedback and have made a number of edits to this section.  In particular, we edited the text to reflect the first-line ART recommendations (two NRTIs and one NNRTI) prior to the WHO recommendation in Dec 2018 transitioning to the integrase inhibitor, dolutegravir. The 2011 guidelines mentioned EFV or NVP in 3 first line regimens (along with either TDF or AZT or d4T as the first agent in the combination therapy). In 2014, TDF/3TC/EFV was the preferred first-line regimen for adults, and this has been stated. Relevant references have been included.

 We disagree as that the Nanda reference (citation numbers have been changed) is not applicable to the statement you indicated. The final conclusion in the Nanda paper is that “More well-designed prospective studies are needed to examine potential drug interactions between ARVs and all contraceptive methods, to better inform guidelines and counseling for the more than sixteen million women living with HIV” and a shorter version of this statement appears in the abstract.

 Methods: with respect to knowing about visits, we know who was seen in CCCs within the dates in their medical charts and the presence of their charts in the record rooms. We have clarified where dates of medical tests etc were also extracted in the revised version. We also added a short description about how pregnancy was typically noted in records and changed “hospital” to “facility” throughout the manuscript.

 We moved the Data Cleaning section to the results. The number of women who switched either ART or implants or both during the observation period are described in the second paragraph of demographic and ART information. We added mean and median durations of observation.

 We censored the observation time after the recommended duration of implant use so as not to confuse the interpretation of results. If women concurrently using “expired” implants and ART experience a pregnancy, one could rightly assume that the failure is due to the loss in implant efficacy. We wanted to ensure that contraceptive failures could only be due to drug-drug interactions and not linked to the devices’ efficacy. While understanding the real world effectiveness issues of women using implants past their labeled effectiveness is interesting, it merits a different study.

 On the sample size: while we had determined in our protocol that we would need at least 177 concurrent users of LNG and EFV and 116 ETG users and EFV to counter the null hypothesis. However, we were concerned that selectively abstracting medical records from the facilities would lead to bias, so instead abstracted all records of concurrent users, yielding a much higher sample. This is why we omit discussion of sample sizes in the manuscript.

 As discussed in the methods, we divided concurrent use for each combination of drugs, instead of assessing use by each woman… We based the original analysis on the regimen on date of pregnancy noted in records. Based on this comment, we reviewed the data and found two women that had a regimen switch within 9 months of the pregnancy date. Both were analyzed as EFV-users, but had switched from a NVP-based regimen within 4-5 months of the date pregnancy was noted. Thus, it is possible these were in fact pregnancies that occurred during NVP use. We have added a note about this in the results.

 We did not use double entry, however field supervisors reviewed case files of women where a pregnancy was detected, as well as random samples of other case files.

 Results: The results section is rather short (though has expanded in this version). There are already 4 subheadings. We hope our re-writing has helped clarify any confusion.

 Table 3: we edited the table to refer to PI-only, not non-NNRTI. Specifically these included LPV/r or ATV/r. Our raw data set included over a dozen combination of anti-retroviral agents (some of which were not consistent with HIV guidelines). A table listing all the possible combination would be unwieldly and contain small numbers for many categories. Furthermore the “PI” category is made up of a small number of observations. We don’t feel breaking this category further is warranted given that we are primarily interested in NVP and EFV. However, researchers interested in other combinations can contact us for the data.

 The discrepancy between Table 3 and the text when it comes to the reference category has been corrected.

 The referent combination was ETG-NVP, not ETG-EFV; the reason for this selection was presumed drug-drug interaction based on previous evidence. We felt comparing all combinations was most efficient. The pregnancy incidence rates remain the same even if the analysis separates by implant types. However, we redid an analysis of pregnancy IRRs for LNG-combinations and inserted the results in the text to address your comment. We didn’t repeat the ETG analysis as the results are the same as shown. The text has been modified to clarify the comparisons. Also, we made Table 3 consistent with figure 1 and removed Figure 2.

 Discussion: You asked us to elucidate why we found no significant difference in the incidence of pregnancies among women using nevirapine and efavirenz-based regimen. We acknowledge the possibility that this finding is an anomaly. In terms of biases, we listed extensive limitations to our approach, with numerous potential errors. However, we doubt that all 40 pregnancies could be ascribed to data errors. Only a little over 30% of the observations included nevirapine, so the comparisons are not balanced. We have spent time exploring our data set to identify possible alternative explanations without seeing any patterns that would provide a plausible explanation.

 Our authorship team is composed of primarily program implementers with an implementation science lens. Identifying pharmacologists to consult with about our results is therefore difficult. We attempted to do so through contacts at Johns Hopkins University without success. With our already prolonged timeline for posting a new version of the paper, we abandoned the effort. We suggest that the findings be included in the literature as an impetus to further study the issue.

 First paragraph: We had constricted this section in one of our versions, but have edited to be explicit about the results for each combination category. We refined our description of the finding related to NVP.

 Second paragraph: We added a sentence acknowledging that a different approach might have yielded even higher numbers of pregnancies. We feel we would be over-reaching if we attempted to comment on pregnancy rates in this context versus in non-African settings, as the number of studies is still very small.

 Regarding the change in the sensitivity analysis removing older women: in general, we found a lack of robustness between preliminary analysis and subsequent cleaning of the data. These pregnancies remain relatively rare events.  However, we may be able to hypothesize that younger women are more fertile than women aged 35-49, and thus the higher fertility negates any differences in type of implants.

 Implications for practice: We have revised the discussion section to reflect the transition to DTG, citing relevant literature. We believe the last sentence of the implications paragraph is already advocating for person-centered care and ability to pick a regimen that fits their fertility intentions.

 Choice of “unintended pregnancy” vs “failure” terminology: We have reviewed our manuscript with this comment in mind and replaced the unintended pregnancies term in some places, as we mostly discuss implant contraceptive failures. However, the measurement of that failure is a pregnancy while using an implant. Therefore there remain a few instances where the term “unintended pregnancy” cannot be replaced.

# Peer review: Reviewer response for version 1

**DOI:** 10.21956/gatesopenres.14082.r27315

**Published:** 2019-07-15

**Authors:** Kavita Nanda

**Affiliations:** 1 Contraceptive Technology Innovation, Global Health, Population and Nutrition, FHI360, Durham, NC, USA

This is an important study that adds to the literature on the issue of contraceptive effectiveness for implants among women taking antiretroviral therapies. However, the study is limited by its retrospective nature, and by the existing limitations of databases designed for other purposes. Of most concern is the outcome ascertainment, as detailed in the specific comments below. 

 Introduction:

- The introduction is very long. Some of it could be moved to the discussion.

Methods:

1. How many research assistants abstracted data for each participant? How was data accuracy monitored?
2. The study inclusion criteria are not clear. Did they have to be currently using an implant to be included? Or were women included if they ever used an implant and ART concurrently?
3.  For what proportion of women were the data incomplete? Did RAs consult other records for all women? Or only those on certain regimens? How were the MSI data matched to the ART data? How was the CCC number matched to other records?
4. How were data collected on reasons for removal? Were actual pregnancies verified if a woman said her implant was removed due to pregnancy? My concern is that a woman could be concerned about the risk of pregnancy and ask for her implant to be removed without actually being pregnant. Also, how good were data on implant removal (for other reasons). 
5. You state that the investigators abstracted 1,612 records from women concurrently using ART and an implant. However, earlier you stated that the RAs abstracted data. Please clarify. Also, are these all women who at any time used ART and an implant concurrently?
6. Please provide details on how you estimated the estimated fertilization date.
7. How good were data on type of implant? Was implant type always noted?
8. The exclusion of pregnancies within three months of implant insertion is important and appropriate.
9. In the discussion of limitations, the author should spend more time discussing the lack of information on other medications, as well as any uncertainties in outcome ascertainment. I am concerned that the way pregnancy was determined may not be accurate, as pregnancies were not clinically verified (just a desire for removal due to pregnancy). I'm also concerned that other co-administered medications are not adequately captured. Retrospective studies such as this, though hypothesis generating, are subject to bias and confounding. Were RAs as diligent in seeking out possible pregnancy in women not using ART?

I confirm that I have read this submission and believe that I have an appropriate level of expertise to confirm that it is of an acceptable scientific standard, however I have significant reservations, as outlined above.

# Response

**Published:** 2019-12-21

**Authors:** Anne Pfitzer

**Affiliations:** Jhpiego, USA

Thank you for your thoughtful review and feedback.

 Introduction: Your comment is consistent with other reviewers and we have reduced the length of the introduction.

 Methods: The first line of the data collection section clearly states that eleven RAs abstracted data (however, the data in the final analysis set excludes data abstracted by two of the RAs.)

 Accuracy was monitored during regular supervision visits by two co-investigators. We moved the sentence at the end of the first section of the Methods to the data collection section in hopes it flows more logically. We added a sentence about the study inclusion criteria in hopes of making clearer. The women did not have to be using an implant during data collection, but at some point between Jan 2011 and Dec 2015 and receiving ART at the same time.

 Regarding incomplete data: most CCC records were missing data about co-morbidities, viral load, and many were missing date of removal, reason for removal, etc. It would difficult to give a proportion as we did not calculate across all variables. And yes, the RAs consulted all medical records of women meeting study eligibility criteria of a CCC visit during the study period. However, we described excluding records with data vital for our analysis in the Data Cleaning section. The most common reason a record was dropped due to incomplete data was because they didn’t have an insertion date for their implant. RAs focused their search of other records for women whose CCC charts were incomplete. It is possible they searched even harder when they suspected there was a contraceptive failure.

 We have modified the statement about MSI data to clarify that this was an aid in searching records but did not dramatically change the process. RAs still confirmed data from client records within the facility, but may have benefited from retrieval of FP client record numbers to do so.

 The RAs used a few key personal identifiers to match records from CCC with those of the same clients in other units (such as ANC). These identifiers were name, age or DOB, home sub-location. RAs were instructed to maintain confidentiality of those details and destroy them after completing medical record abstraction and having those reviewed by a supervisor.

 Data on reasons for removal were abstracted from case files with the rest of the information recorded in a woman’s record. The abstraction form included check boxes for likely reasons: pregnancy/method failure, menstrual/bleeding issues, desire for pregnancy, partner/spouse request, and expiration. Another field allowed RAs to add comments or provide an alternative reason for removal if one was found in the records. Almost all women with a recorded date of removal also had an indicated reason.

 Due to the nature of the study design, RAs could not verify pregnancies. However, missing or inconclusive data were found in the client’s records in the Comprehensive Care Clinics, RAs referred to other sources (e.g., FP registers, maternity ward registers, provider notes) to verify and complete the abstraction details.

 Three of the investigators interacted closely with providers. We have never heard of a case of a client asking for a removal out of concern of a risk of pregnancy that then later turned out not to have been. Providers are aware of amenorrhea as a possible side effect of implants. They also routinely carry out pregnancy tests.

 Thank you for pointing out an inconsistency in our writing about the role of RAs as opposed to investigators. We have corrected. Research assistants abstracted the data under the investigators supervision (including close supervision of two of the co-authors of this paper).

 Regarding concurrent use, no, some of the women whose data were abstracted did not use an implant and ART concurrently. Some used both, but not at the same time and were dropped during data cleaning (they’re included in the group that had fewer than three months of concurrent use)

 We did not estimate the fertilization date.  We only documented pregnancy status from clinical records. However, we also added content in the limitations section to explain that records were not always clear.

 About the quality of data on implant types: we have added detail on the process RAs undertook to clarify type of implant. We excluded any records where the RAs were unable to determine the type of implant. We considered whether implant type was missing in data cleaning. After other cleaning, 1 person was dropped for not having implant type recorded. However, records with other missing information may have been cleaned prior to that step.

 Thank you for the affirmation about excluding pregnancies within three months of implant insertion.

 Discussion and limitations: We are limited by the way health providers ascertain a pregnancy and record pregnancy in the charts. We have added this in the limitations section of the discussion 

 In terms of the concern related to other medications, we agree with you as this was our secondary research objective. However, record-keeping on other medications was very poor and we were not able to analyze this. But you will note that we expanded the results and discussion around potential drug-drug interactions further compounded with TB treatment, in response to reviewer comments. We already noted this in the second paragraph of our limitations section, but have expanded on it in this version.

 Regarding pregnancy in women not on ART. We do not include a comparison of women not on ART.

# Peer review: Reviewer response for version 1

**DOI:** 10.21956/gatesopenres.14082.r27364

**Published:** 2019-07-01

**Authors:** Catherine A. Chappell

**Affiliations:** 1 Department of Obstetrics, Gynecology and Reproductive Sciences, School of Medicine , University of Pittsburgh, Pittsburgh, PA, USA

This is a retrospective cohort study of pregnancy rates among Kenyan women living with HIV on antiretroviral therapy and using the subdermal implantable contraceptive implant. The data is presented is a clear fashion, however some significant limitations of the study design precludes the reliability of the data.  

1. Abstract: The background is too long and the conclusion is too short. For example, noting the percentage of women who are married in the cohort is too granular for the abstract. The background should focus on why ART might impact the contraceptive efficacy of the implant. 
2. Introduction: The introduction is too long. Recommend giving only relevant background, such as why there might be a contraceptive-drug interaction and the number of women globally and in Kenya are using ART and implants together. What is the knowledge gap this study fills?  
3. Introduction, paragraph 3, sentence 2: I do not understand the relevance of marital status in reporting the contraceptive implant use. More importantly would be how many Kenyan women use the contraceptive implant. 
4. Introduction, paragraph 4, sentence 4: The interpretation of citation 20 is inaccurate, unless the authors have communicated directly with the authors of citation 20. In this study only 25 women were on NNRTIs, but they did not differentiate whether the NNRTI was EFV or NVP.  
5. Introduction, paragraph 4: Recommend including PK study by Chappell *et al.* (2017) ^1^ which showed ETG PK changes with EFV, but not NVP. Also Scarsi KK, *et al.,* citation 21 has been published in CID. Please update the reference.
6. Methods: The section entitled “data cleaning” and Table 1 usually go into the results section.  
7. Results, section “Analysis of incidence by ART and contraceptive use”, paragraph 1, sentence 4: This sentence is confusing because of the way it is written. I would recommend stating more clearly that LNG implant users were less likely to become pregnant than ETG implant users. 
8. Results, section “Analysis of incidence by ART and contraceptive use”, paragraph 2: I believe in the table the reference population is ETG-NVP. 
9. Results, last paragraph, sentence one: What pregnancy incidence is this referring to? The overall pregnancy incidence among women age 15-34? Or just those on NNRTIs? 
10. Results, last paragraph, sentence 2: The findings are unclear. Could these data be placed into a table for clarity?  
11. Discussion, limitations: The largest limiter is the lack of ability to know if any of these women were on medications for TB. My concern is that the women on NVP-based ART, had HIV longer and more likely had lower CD4 counts, and thus were more likely to get TB and be on rifampicin. Do you have any idea how many women had TB over the study period? How certain are you that the women on NVP were actually on NVP and had not been switched to EFV for some times depending on ART availability. The finding of the increased pregnancy rate among women on NVP using contraceptive implants is contradictory to all the published literature (though limited). In the limitation section, I recommend raising these concerns regarding the accuracy of the data and cautions readers on interpretation.  

I confirm that I have read this submission and believe that I have an appropriate level of expertise to confirm that it is of an acceptable scientific standard, however I have significant reservations, as outlined above.

## References

[1] : Efavirenz decreases etonogestrel exposure. *AIDS*.2017;31(14) : 10.1097/QAD.0000000000001591 1965-1972 10.1097/QAD.0000000000001591 PMC557887128692531

# Response

**Published:** 2019-12-21

**Authors:** Anne Pfitzer

**Affiliations:** Jhpiego, USA

Thank you for your review.

 We appreciate the feedback on the abstract and have shortened the abstract background section. With the remaining word count, we expanded on key results. We also shortened the introduction (and re-organized it as a result of another reviewer’s feedback). We removed the distinction about married and unmarried women, although this required doing our own analysis of survey data as it is common in the family planning literature and FP data reports such as PMA to refer separately to married women and sexually active unmarried women. However, you are right that marital status is not pertinent here and we have revised. Our analysis of PMA’s dataset shows that of 5776 women of reproductive age (15-49, like our study), 2500 currently using a method of FP. Of those, 813 used an implant.

 Introduction, paragraph 4, sentence 4: You are correct that the distinction is just between “HAART with PI” and “HAART NNRTI”.  However, this is no longer relevant as we’ve removed this portion of the text. We also appreciate pointing us in the direction of the Chappell paper in AIDS. We added it to the discussion section and have also updated the Scarsi reference.

 Methods: We moved the data cleaning section to the results per your recommendation.

 Results, section “Analysis of incidence by ART and contraceptive use”: we appreciate the suggestions and corrections and have incorporated them. We also hope we have clarified what was previously the last paragraph. And regarding that paragraph, you made a comment referring to the sensitivity analysis (which has now incorporated into Table 3 per another reviewers suggestion). We have tried to reword the description of the analysis and its interpretation to make it clearer. The main purpose of such an analysis is to describe the robustness of the results. Our analysis showed lack of robustness in the difference in failures by type of implant. We don’t feel showing the detailed data adds value.

 Discussion: The multiple reviewer comments about the role of TB medications inspired to take a closer look at the subset of records with indications of TB.  We added a paragraph on TB results and a new box summarizing our exploration of a subset of records with TB treatment that describes: the number or percentage of records with documentation of TB treatment in clinical records. We have added more commentary on the availability of TB-related data in the medical charts in the discussion.

 With respect to ascertaining that “women on NVP were actually on NVP and had not been switched to EFV for some times depending on ART availability”. Our methods for data abstraction relied upon complete and timely documentation within the facility-based comprehensive care clinic records. If there was a separate set of TB clinic client records, we did not review them. As for regimen, we can only attest that providers recorded the regimen of clients on their “Blue card” at every visit. It seems unlikely that an error of regimen would recur over multiple visits. We also did not hear of any historical concerns about ART availability.

 We agree with you about the contradiction of our NVP finding with the literature. We stated this at the end of the first paragraph of the discussion. We added an acknowledgement at that point that this may be an artifact of the data quality and limitations of chart reviews.

 We have included 3 paragraphs of limitations in the paper and believe we are transparent on the problems with the chart review.
